# Effects of cadmium and monensin on renal and cardiac functions of mice subjected to subacute cadmium intoxication

**DOI:** 10.2478/intox-2014-0015

**Published:** 2014-11-15

**Authors:** Juliana Ivanova, Yordanka Gluhcheva, Sonja Arpadjan, Mariana Mitewa

**Affiliations:** 1 Faculty of Medicine, Sofia University “St. Kl. Ohridski”, Kozjak 1 Str., 1407 Sofia, Bulgaria; 2 Institute of Experimental Morphology, Pathology and Anthropology with Museum, BAS, Acad. Georgi Bonchev Str., bl. 25, 1113 Sofia, Bulgaria; 3 Faculty of Chemistry and Pharmacy, Sofia University, “St. Kl. Ohridski” J. Bourchier blvd 1, 1164 Sofia, Bulgaria

**Keywords:** Cadmium, monensin, *in vivo* model, renal function, cardiac function, chelating agents

## Abstract

Cadmium (Cd) is a well-known nephrotoxic agent. Cd-induced renal dysfunction has been considered as one of the causes leading to the development of hypertension. The correlation between Cd concentration in blood and urine and cardiovascular diseases has been discussed in many epidemiological studies. A therapy with chelating agents is utilized for the treatment of toxic metal intoxication. Herein we present novel information indicating that monensin (applied as tetraethylammonium salt) is a promising chelating agent for the treatment of Cd-induced renal and cardiac dysfunction. The study was performed using the ICR mouse model. Adult ICR male mice were divided into three groups with six animals in each group: control (received distilled water and food *ad libitum* for 28 days); Cd-intoxicated (treated orally with 20 mg/kg b.w. Cd(II) acetate from day 1 to day 14 of the experimental protocol), and monensin treated group (intoxicated with Cd(II) acetate as described for the Cd-intoxicated group followed by oral treatment with 16 mg/kg b.w. tetraethylammonium salt of monensic acid for 2 weeks). Cd intoxication of the animals resulted in an increase of the organ weight/body weight indexes. Cd elevated significantly creatinine and glucose level in serum. Monensin treatment improved the organ weight/body weight ratios. The therapy of the Cd-intoxicated animals with monensin ameliorated the creatinine and glucose level in serum and decreased the concentration of the toxic metal ions in the heart and kidneys by 54% and 64%, respectively.

## Introduction

Cadmium is a toxic element. When it enters the bloodstream, it binds to albumin and other high molecular weight proteins. It is then transferred to the liver (Nordberg, 2004). The accumulation of Cd in the liver triggers synthesis of metallothionein (MT), which has been considered a primary defensive mechanism against Cd-intoxications (Nordberg & Nordberg, 2004). The Cd-MT complex passes through the glomeruli and is taken up by the renal tubular cells (Nordberg *et al.*, 1994). In the kidneys, this complex is destroyed. MT is catabolized in the lysosomes and Cd is released (Nordberg *et al.*, 1994). The accumulation of Cd in the kidneys causes damage of the renal proximal tubules. Injury of the renal proximal tubules was observed in animals exposed to Cd in environmentally relevant doses, demonstrating the necessity of developing an effective therapy for the treatment of Cd-intoxications (Thijssen *et al.*, 2007). The renal dysfunction induced by Cd has been considered one of the causes for the development of hypertension (Satarug, 2005). The effect of Cd on the vascular system and cardiac function was discussed by Sompamit *et al.* (2010), Prozialeck *et al.* (2006, 2008), Molloaoglu *et al.* (2006), Manna *et al.* (2008), and Donpunha *et al.* (2011). Furthermore, the correlation between blood and urine Cd concentration and diseases such as idiopathic dilated cardiomyopathy (Smetana *et al.*, 1987), peripheral arterial disease (Nordberg, *et al.*, 1994), stoke, heart failure, atherosclerosis was documented in many epidemiological studies (Tellez-Plaza *et al.* (2010), Peters *et al.* ( 2010), Ross (1999).

Different chelators were shown to decrease Cd concentration in the kidneys of animals subjected to Cd-intoxication, yet they were not found to be very effective in reducing Cd concentration in other organs (Blanusa *et al.*, 2005; Flora & Pachauri, 2010; Andujar, 2010). Furthermore, most of the chelating agents were administrated *s.c.* or *i.p.*, which might cause severe side effects (Blanusa *et al.*, 2005; Flora & Pachauri, 2010). Of the oral chelating agents DMSA (2,3 dimercaptosuccinic acid) has been used in the therapy of heavy metal intoxication. However, this chelating agent is hydrophilic and does not excess the toxic metal ion accumulated in the intracellular space (Flora & Pachauri, 2010). Furthermore, the effect of DMSA on Cu homeostasis should be considered when this agent is utilized for the treatment of heavy metal intoxications in humans (Flora & Pachauri, 2010). Recent studies on animal models showed that the polyether ionophorous antibiotic monensin is much more effective than DMSA in the therapy of lead (Pb) intoxication (Hamidinia, *et al.*, 2006). In our previous paper we reported that this antibiotic reduced the concentration of Cd in organs of mice subjected to subacute Cd intoxication (Ivanova, *et al.*, 2012). We found that monensin did not disturb the homeostasis of Cu and Zn. Furthermore, the antibiotic significantly improved Fe metabolism in mice subjected to subacute Cd intoxication (Ivanova *et al.*, 2012). These promising results indicated that the possible application of monensin as a chelating agent for the therapy of Cd-intoxication should be studied in detail.

The present study was designed to assess the effect of monensin on Cd-induced renal dysfunction and cardiac impairment.

## Materials and methods

### Animal model

Sixty-day-old adult male ICR mice were purchased from the Animal Care Unit Slivnica (Bulgaria). They were housed at the Institute of Experimental Morphology, Pathology and Anthropology with Museum (Bulgarian Academy of Sciences, Sofia) under conventional conditions at room temperature with 12 h light/12 h dark cycle and controlled humidity. The animals were divided into three groups with six mice each. The first (control) group received standard diet and had free access to distilled water during the experimental protocol. The second group of animals (Cd-treated) was exposed to 20 mg/kg body weight Cd(CH_3_COO)_2_ × 2H_2_O in drinking (distilled) water once daily for two weeks. During the following 14 days of the experiment, the animals from this group received distilled water and food *ad libitum*. The third group (monensin-treated mice) was administrated Cd(CH_3_COO)_2_ × 2H_2_O as described above, followed by treatment with tetraethylammonium salt of monensic acid (16 mg/kg body weight in distilled water) during the days 15 to 28 of the experiment. On day 29 of the experimental protocol, all animals were sacrificed under light ether anesthesia and the samples were collected for analysis. The organs were stored at –20 °C prior to analysis. Blood samples were collected in heparinized tubes, centrifuged, and the resulting plasma samples were stored at –20 °C. The animal studies were approved by the Ethics Committee of the Institute of Experimental Morphology, Pathology and Anthropology with Museum, BAS.

### Chemicals

The sodium salt of monensin was a gift by Biovet Ltd. (Peshtera, Bulgaria). Tetraethylammonium hydroxide (Et_4_NOH), nitric acid (HNO_3_), and diethyl ether (Et_2_O) were purchased from Merck (Darmstadt, Germany).

### Preparation of monensic acid

Monensic acid A monohydrate was prepared from sodium monensin (711 mg, 1 mmol) by applying the procedure previously described (Ivanova *et al.*, 2010).

### Biochemical analysis

The biochemical analysis was performed in the clinical laboratory “Ramus” (Sofia, Bulgaria) using established analytical protocols. The laboratory “Ramus” is certified by the Ministry of Health (Sofia, Bulgaria) to perform clinical analyses.

### Atomic absorption analysis

The organs were digested with concentrated HNO_3_ (free of metal ions), as described previously (Ivanova *et al.*, 2012). The determination of Cd in the kidneys was performed by a flame (Perkin Elmer Analyst 400, air–acetylene flame) atomic absorption spectrophotometer. The analysis of Cd in the hearts of the animals was conducted on an electrothermal analyzer (Zeeman Perkin Elmer 3030, HGA 600). Certified Reference Materials from the International Atomic Energy Agency (IAEA-H-8 (kidney) and IAEA-H-4 (animal muscle) were applied to control analytical accuracy.

### Statistical calculations

The results for the three study groups are presented as the mean value ± SD (*n*=6 for each group). Student's *t*-test was applied to determine the significance of the differences between the experimental results of two groups. The difference between two groups was considered significant at *p*<0.05.

## Results and discussion

**Figure 1:**
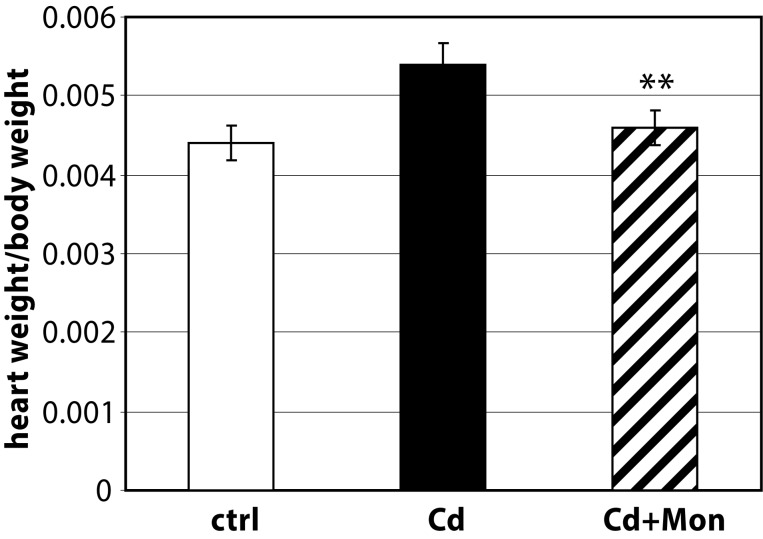
Heart weight/body weight ratio of the experimental animals. Each column represents mean±SD, *n*=6. Asterisk (*) represents significant differences between the Cd-treated group and normal controls (*p<*0.05); Double asterisk (**) represents significant differences between the monensin-treated group and the Cd-intoxicated animals (*p<*0.05)

**Figure 2:**
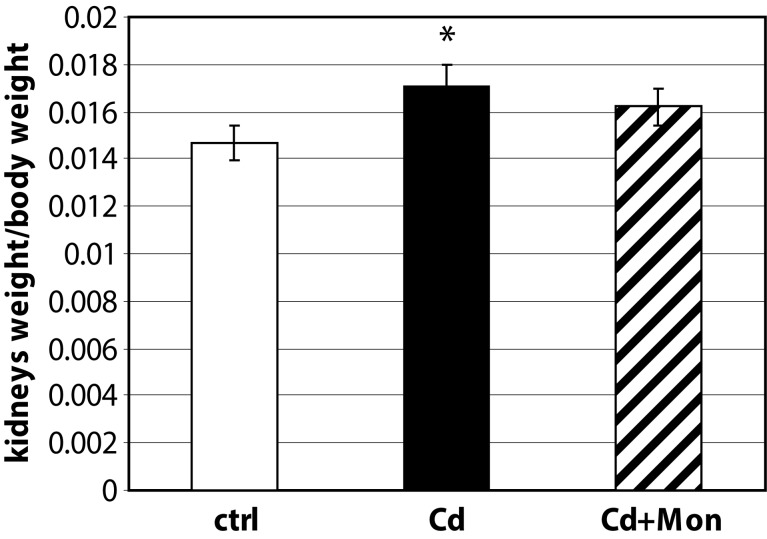
Kidney weight/body weight ratio of the experimental animals. Each column represents mean±SD, *n*=6. Asterisk (*) represents significant differences between the Cd-treated group and normal controls (*p<*0.05);

In this study we present novel data regarding the effect of monensin on the kidney and heart weight of Cd-intoxicated animals (see Figures 1 and 2).

The administration of Cd(II) acetate to mice over 2 weeks resulted in a significant elevation of the heart weight by 20% compared to the controls (*p<*0.05). The weight of the kidneys was also affected by Cd intoxication. The Cd-induced organomegaly has been associated with inflammation processes, triggered by this metal (Donpunha *et al.*, 2011). Treatment of the Cd-intoxicated mice with monensin returned the weight of the heart to normal. Monensin decreased also the weight of the kidneys in the Cd-treated animals, but the difference between Cd-intoxicated and monensin detoxicated groups was not statistically significant. The data showed that the effect of monensin on Cd-induced cardiac toxicity was more pronounced compared to the alterations in renal function triggered by Cd.

**Figure 3:**
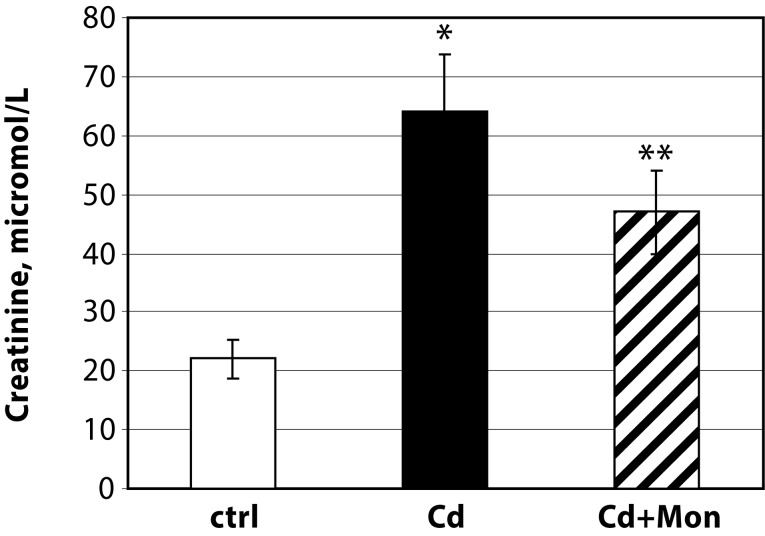
Creatinine concentration in serum of experimental animals. Each column represents mean±SD, *n=*6; Asterisk (*) represents significant differences between the Cd-treated group and normal controls, *p<*0.05; Double asterisk (**) - significant differences between monensin-treated group and the Cd-intoxicated animals (*p<*0.05)

The biochemical studies showed that Cd increased creatinine in the serum of Cd intoxicated animals (Figure 3). Our finding supports the conclusion that serum cretinine concentration could be used to monitor cadmium-induced renal injury (Bharavi *et al.*, 2011). The decrease of the level of creatinine in monensin-treated mice by 30% (*p<*0.05) confirmed the protective effect of this antibiotic on renal function of mice exposed to subacute Cd intoxication.

**Figure 4:**
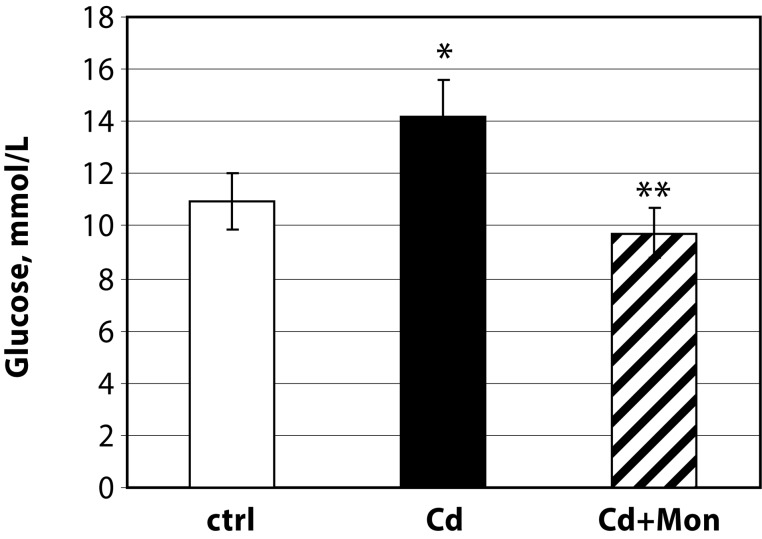
Glucose concentration in serum of the experimental animals. Each column represents mean±SD, *n=*6; Asterisk (*) represents significant differences between the Cd-treated group and normal controls, *p<*0.05; Double asterisk (**) - significant differences between the monensin-treated group and the Cd-intoxicated animals (*p<*0.05)

Treatment of the animals with Cd(II) acetate led to a significant increase of glucose concentration in serum (Figure 4). Different cellular and physiological mechanisms have been proposed for Cd-induced elevation of glucose level in serum (Edward & Prozialeck, 2009). Animal studies performed by Edward and Prozialeck (2009) demonstrated a direct effect of Cd on the pancreas. Cd was found to alter insulin release from β-pancreatic cells. *In vitro* studies on mouse renal cortical cells showed that Cd decreased both glucose uptake and expression of SGLT1, an Na^+^-dependent glucose symporter (Blumenthal *et al.*, 1998). These results corroborated the studies on Cd-treated rats showing that Cd elevated activities of enzymes responsible for gluconeogenesis in kidney tissue (Chapatwala, *et al.*, 1982). Treatment of the Cd-intoxicated mice with monensin in the present study attenuated the Cd-induced increase of glucose concentration in serum.

**Figure 5:**
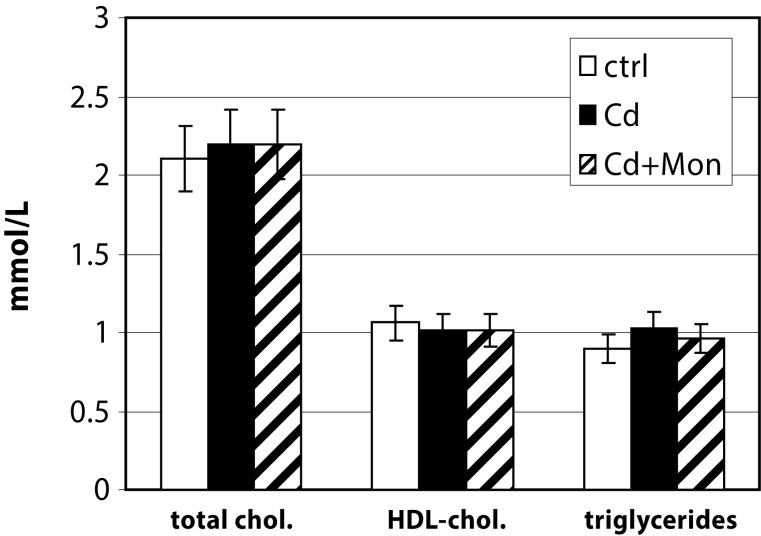
Lipid profile in serum of the experimental animals. Each column represents mean±SD, *n=*6; Asterisk (*) represents significant differences between the Cd-treated group and normal controls, *p<*0.05; Double asterisk (**) - significant differences between the monensin-treated group and the Cd-intoxicated animals (*p<*0.05)

Figure 5 presents the results from the biochemical analysis of the lipid profile in the three groups of mice studied. The lipid profile (total cholesterol, HDL-cholesterol and triglycerides) was not affected either by cadmium treatment or by monensin therapy. The study by Messner *et al.* (2009) demonstrated that the plaque formation induced by Cd did not always correlate with alteration of the lipid profile. The authors concluded that Cd exerted its atherogenic activity by causing endothelial damage (Messner *et al.*, 2009).

Histological studies on cardiac and renal tissues revealed that treatment of Cd-intoxicated mice with monensin significantly improved the morphology of both organs studied (data not shown).

**Figure 6:**
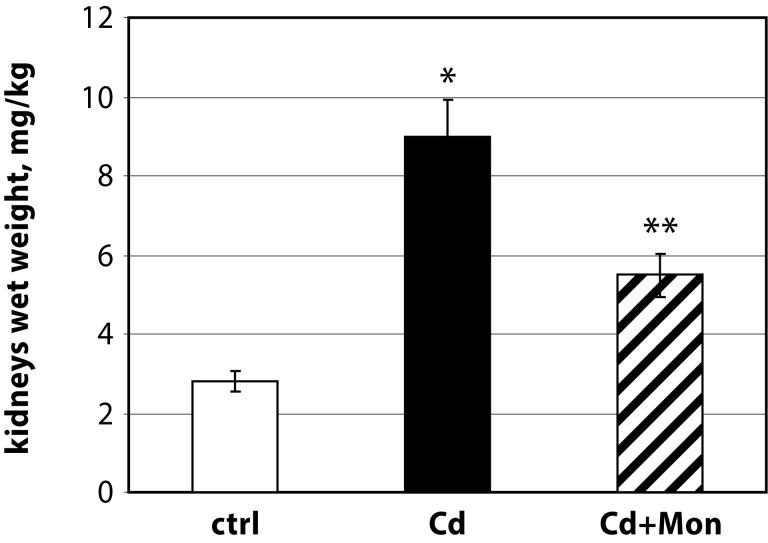
Cd concentration in kidneys of the experimental animals. Each column represents mean±SD, *n=*6; Asterisk (*) represents significant differences between the Cd-treated group and normal controls (*p<*0.05); Double asterisk (**) represents significant differences between the monensin-treated group and the Cd-intoxicated animals (*p<*0.05)

**Figure 7:**
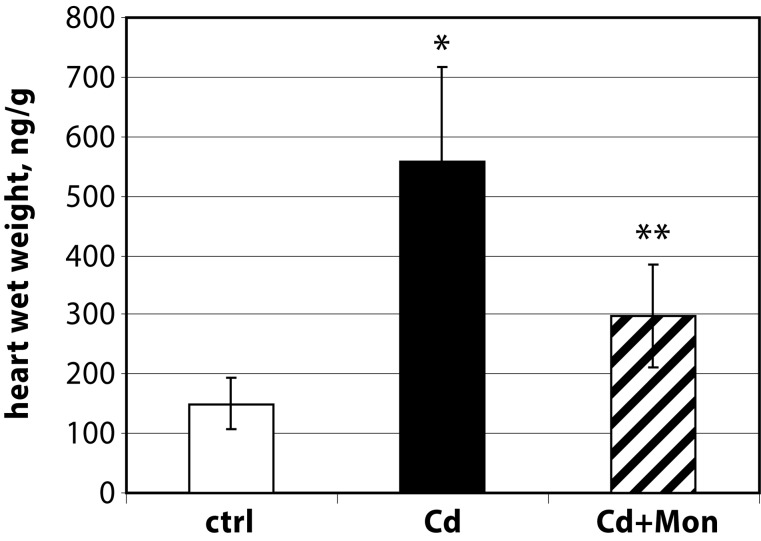
Cd concentration in the heart of the experimental animals. Each column represents mean±SD, *n=*6; Asterisk (*) represents significant differences between the Cd-treated group and normal controls (*p<*0.05); Double asterisk (**) represents significant differences between the monensin-treated group and the Cd-intoxicated animals (*p<*0.05).

The data from the atomic absorption analysis showed that the highest Cd concentrations were measured in the kidneys and hearts of the Cd-treated animals (second group) (Figures 6 and 7). The values for the concentration of Cd in both organs are higher than those reported in our previous study where the animals were subjected to 10 mg/kg Cd(II) acetate daily treatment for 2 weeks (Ivanova *et al.*, 2012). The data presented in this study confirmed that accumulation of Cd in the organs was dose dependent. Monensin decreased the Cd concentration in the kidneys and heart of Cd-intoxicated animals by 57 and 64%, respectively (*p<*0.05). These data are in good agreement with the results from biochemical and histological analyses and support the conclusion that the polyether ionophorous antibiotic monensin could be a promising chelating agent for the treatment of renal dysfunction and cardiac impairment in cases of Cd intoxication.
